# Multi‐Omics Integration Identifies PTGS2 as a Candidate Diagnostic Biomarker and Precise Therapeutic Target Related to *Schisandra chinensis* for Osteoarthritis

**DOI:** 10.1111/cbdd.70368

**Published:** 2026-08-17

**Authors:** Lingtian Min, Yikai You, Shiqi Ren, Cheng Chen

**Affiliations:** ^1^ Department of Orthopedics Nantong Hospital of Traditional Chinese Medicine, Nantong Hospital to Nanjing University of Chinese Medicine Nantong China; ^2^ Department of Rehabilitation Suqian Hospital of Integrated Chinese and Western Medicine Suqian China; ^3^ ZUMRI – Donghai County People's Hospital Joint Lab Zhuhai UM Science and Technology Research Institute Zhuhai China; ^4^ Donghai County People's Hospital ‐ Jiangnan University Smart Healthcare Joint Laboratory Donghai County People's Hospital Lianyungang China; ^5^ Department of Orthopedics, Jiangsu Province (Suqian) Hospital, Suqian First Hospital Nanjing Medical University Suqian China

**Keywords:** biomarker, molecular docking, network pharmacology, osteoarthritis, PTGS2, *Schisandra chinensis*, single‐cell transcriptomics

## Abstract

Currently, there is a lack of effective disease‐modifying drugs for osteoarthritis (OA), and existing treatment methods often accompany significant adverse reactions. *Schisandra chinensis* (SC), as a traditional Chinese medicine with anti‐inflammatory activity, may have therapeutic potential for OA, but its specific molecular mechanism is not yet clear. This study adopts network pharmacology strategy to explore the potential mechanism of SC intervention in OA. Screen SC active ingredients through TCMSP database and obtain OA‐related targets by combining OMIM, TTD, and GeneCards databases. Venn analysis shows that there are only 5 common targets between SC and OA; Further analysis revealed that PTGS2 is the only candidate target with diagnostic value (AUC = 0.702, *p* = 0.015), while AR, ESR1, DPP4, and CHRM2 did not show clinical diagnostic efficacy. Functional enrichment analysis suggests that common targets mainly involve steroid hormone responses and extracellular matrix (ECM) related pathways, with a significant enrichment in “response to steroid hormones” (adjusted *p* = 1.2 × 10 ^−5^), and GSEA also shows activation of the ECM‐receptor interaction pathway (NES = 1.87, *p* = 0.002). The molecular docking results indicate that the SC active ingredients MOL008957 and MOL008978 can form stable binding with PTGS2. The qRT‐PCR results further confirmed that the expression of PTGS2 was significantly downregulated in the OA model after SC intervention. Single cell transcriptome analysis showed that PTGS2 was mainly enriched in monocytes and gradually increased with the pseudo temporal progression; Cell communication analysis revealed that monocytes can interact with NK cells and T cells through the MIF‐CD74 + CXCR4 and MIF‐CD74 + CD44 signaling axes. After virtual knockout of PTGS2, downstream regulatory networks suggest that GSN may be a key target gene, mainly involving collagen containing extracellular matrix and ECM structural components. In summary, SC may exert its anti OA effect mainly by selectively inhibiting PTGS2 and maintaining the homeostasis of ECM related pathways. PTGS2 is expected to become a candidate biomarker for OA and a potential target for SC intervention in OA, but further experimental verification of the specific efficacy and mechanism of SC active ingredients is still needed.

## Graphical abstract

This study used multi‐layer methods such as network pharmacology, differential expression analysis, functional enrichment and GSEA, molecular docking, single‐cell transcriptomics and virtual knockout to analyze the molecular mechanism of *Schisandra chinensis* (SC) in treating osteoarthritis (OA). The results showed that there were only 5 intersecting targets between SC and OA, among which PTGS2 had diagnostic significance (AUC = 0.702, *p* = 0.015). Molecular docking confirmed that SC active ingredients (such as MOL008957, MOL008978) can stably bind to PTGS2; QRT‐PCR further showed that PTGS2 was significantly reduced in the OA + SC group. Single cell analysis showed that PTGS2 was enriched in monocytes and increased with pseudo time progression; cell communication prompts MIF ‐ (CD74 + CXCR4) and MIF ‐ (CD74 + CD44) to connect monocytes with NK cells and T cells. After virtual PTGS2 knockout, downstream key genes GSN are enriched in collagen related and ECM structural component pathways. The research conclusion suggests that the core of SC's anti OA effect lies in selectively inhibiting PTGS2 and stabilizing ECM related pathways. PTGS2 is both a potential therapeutic target and a diagnostic biomarker for SC responsive OA, providing a mechanistic basis for the development of safer and disease‐modifying SC derived OA drugs.

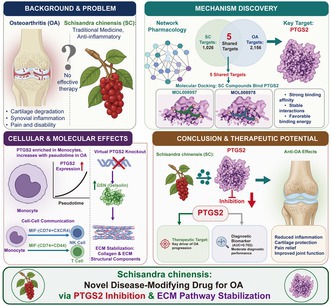

## 1 Introduction

Osteoarthritis (OA), the most prevalent musculoskeletal disorder, affects over 528 million individuals globally, posing a significant public health challenge due to limited disease‐modifying therapeutic options (Bliddal et al. 2024; Cao et al. 2024). Current pharmacological strategies predominantly focus on symptom management using non‐steroidal anti‐inflammatory drugs and corticosteroids, which offer only temporary relief and carry substantial risks of gastrointestinal complications, cardiovascular events, and accelerated cartilage degradation with prolonged use (Courties et al. 2024; Krakowski et al. 2024). The pressing demand for safer, mechanism‐based treatments has heightened interest in traditional medicinal herbs with favorable safety profiles, particularly those capable of targeting multiple pathophysiological pathways without the off‐target effects associated with conventional therapies (Deng et al. 2024; Moseng et al. 2024).

*Schisandra chinensis* (SC), a traditional Chinese medicinal herb recognized for its anti‐inflammatory and antioxidant properties, has demonstrated promising effects in preclinical models of joint disorders (Chen et al. 2024; Luan et al. 2024). Clinical observations indicate that SC extracts may alleviate pain and enhance joint function in patients with OA; however, its molecular mechanisms remain inadequately characterized (Ehambarampillai and Wan 2025; Zhang et al. 2025). This knowledge gap is critical, as SC is composed of over 30 bioactive compounds, including schisandrin, gomisin, and deoxyschisandrin, which may act synergistically on various targets—an intricate complexity that conventional reductionist methodologies cannot address (Meng‐ran et al. 2020; Meng et al. 2025). Recent advancements in network pharmacology present unprecedented opportunities to elucidate such multi‐component, multi‐target interactions through the integration of bioinformatics and systems biology (Cheng, Wang, et al. 2024; Chi et al. 2024; Fan et al. 2024; Zhang et al. 2024).

Emerging evidence implicates prostaglandin‐endoperoxide synthase 2 (PTGS2/COX‐2) as a pivotal mediator of synovial inflammation and pain in early OA, with expression levels closely correlating with disease progression (Zhen et al. 2017; Tanaka et al. 2024; Arnold et al. 2025). However, the relationship between SC's active constituents and PTGS2 regulation in OA pathogenesis remains unexplored. Additionally, while extracellular matrix (ECM) degradation is a hallmark of OA progression, no investigations have addressed SC's potential effects on ECM‐receptor interactions, a pathway increasingly recognized as essential for maintaining cartilage integrity (Li et al. 2013; He et al. 2025; Tang et al. 2025).

This study aims to bridge these critical gaps by employing an integrated network pharmacology and molecular docking approach to systematically identify SC's therapeutic targets in OA and elucidate its mechanisms of action. The hypothesis posits that SC exerts anti‐OA effects through selective modulation of PTGS2 and ECM‐related pathways, thereby providing a mechanistic basis for its clinical efficacy with an improved safety profile compared to non‐selective COX inhibitors. By combining target prediction, expression validation, and structural binding analysis, this research lays a pharmacological foundation for the development of SC‐derived disease‐modifying OA drug (DMOAD) therapies.

## 2 Results

### 2.1 SC Shared 5 Potential Therapeutic Targets With OA‐Related Genes Through Multi‐Database Screening

**FIGURE 1:**
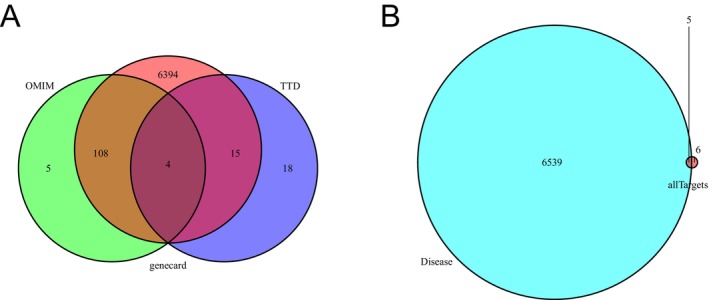
Venn diagram of target screening of *Schisandra chinensis* for osteoarthritis and distribution of disease‐related genes. (A) The Venn diagram shows the overlap of targets among three datasets: OMIM, TTD, and GeneCards. There were 5 overlapping targets between Disease and OMIM, 18 between Disease and TTD, 108 between Disease and GeneCards, and 6394 between Disease and AllTarg. (B) The disease‐related gene set contained a total of 6539 genes, of which 5 were potential targets of *Schisandra chinensis*.

Venn analysis of three target databases (OMIM, TTD, GeneCards) identified a total of 6544 genes associated with OA (Figure 1A). An intersection analysis with the targets of SC revealed five overlapping genes (Figure 1B). These five shared targets represent the core candidate molecules for SC‐mediated intervention in OA, derived from a systematic cross‐referencing of disease‐specific and compound‐target databases. No additional targets outside this overlap were considered for further analysis.

### 2.2 Analysis of Interaction Network, Expression Differences, and Diagnostic Efficacy of Key Targets of SC

**FIGURE 2:**
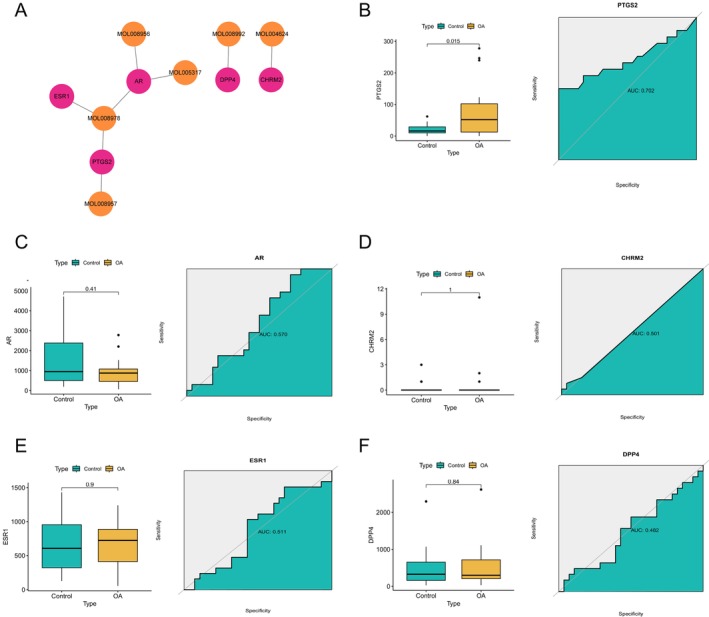
Analysis of the interaction network, expression differences, and diagnostic performance of key targets of *Schisandra chinensis*. (A) Interaction network between the active components of *Schisandra chinensis* (orange nodes, including MOL008956, MOL008992, MOL004824, etc.) and key targets (pink nodes: AR, PTGS2, ESR1, DPP4, and CHRM2), demonstrating the potential interactions between the active components and the targets. (B) Expression difference of PTGS2 between the osteoarthritis (OA) and control groups (box plot, *p = 0.015) and receiver operating characteristic (ROC) curve analysis (AUC = 0.702), suggesting that PTGS2 may serve as a potential diagnostic marker for OA. (C) Expression difference of AR between the OA and control groups (box plot, *p* = 0.41) and ROC curve analysis (AUC = 0.570), showing no significant difference in AR expression between the two groups and relatively low diagnostic performance. (D) Expression difference of CHRM2 between the OA and control groups (box plot, *p* = 1) and ROC curve analysis (AUC = 0.501), indicating no significant change in CHRM2 expression in OA and limited diagnostic value. (E) Expression difference of ESR1 between the OA and control groups (box plot, *p* = 0.9) and ROC curve analysis (AUC = 0.511), suggesting that the expression and diagnostic significance of ESR1 in OA are not significant. (F) Expression difference of DPP4 between the OA and control groups (box plot, *p* = 0.84) and ROC curve analysis (AUC = 0.482), showing no statistically significant difference in DPP4 expression or diagnostic performance in OA.

Among the key targets of SC in patients with OA, PTGS2 was the only significantly dysregulated and diagnostically relevant target. The protein‐compound interaction analysis confirmed binding between active components of SC (e.g., MOL008956, MOL008992, MOL004824) and key targets (AR, PTGS2, ESR1, DPP4, CHRM2) (Figure 2A). Differential expression analysis indicated that PTGS2 was significantly upregulated in patients with OA compared to controls (*p* = 0.015; Figure 2B), with an area under the receiver operating characteristic (ROC) curve (AUC) of 0.702, suggesting moderate diagnostic utility. In contrast, AR demonstrated no significant expression difference (*p* = 0.41; Figure 2C) and low diagnostic value (AUC = 0.570). Similarly, CHRM2 (*p* = 1.0; AUC = 0.501; Figure 2D), ESR1 (*p* = 0.9; AUC = 0.511; Figure 2E), and DPP4 (*p* = 0.84; AUC = 0.482; Figure 2F) showed no statistically significant dysregulation or diagnostic efficacy in OA. Thus, PTGS2 emerged as the sole target indicating both significant OA‐associated dysregulation and clinically relevant diagnostic potential.

### 2.3 SC Targets Were Predominantly Enriched in Steroid Hormone Response, Nuclear Receptor Signaling, and Synaptic Membrane‐Related Functions

**FIGURE 3:**
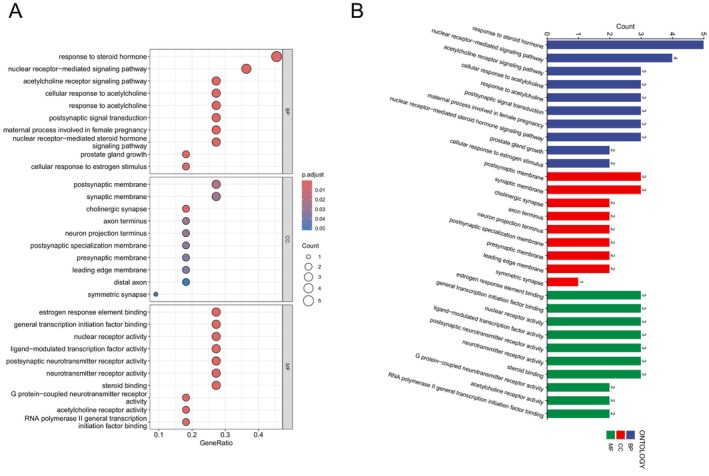
Gene Ontology (GO) Functional Enrichment Analysis of Potential Targets of *Schisandra* chinensis in the Treatment of Osteoarthritis. (A) Bubble plot showing GO enrichment results across three categories: Biological process (BP), cellular component (CC), and molecular function (MF). The *x*‐axis represents the GeneRatio (the ratio of enriched genes to the total number of input genes), bubble size indicates the number of enriched genes (Count), and the color gradient reflects the adjusted *p*‐value (*p*.adjust), with red indicating higher significance (smaller *p*.adjust values) and blue indicating lower significance (larger *p*.adjust values). In the BP category, “response to steroid hormone” and “nuclear receptor‐mediated signaling pathway” exhibited the highest GeneRatio and strongest enrichment. In the CC category, “postsynaptic membrane” and “synaptic membrane” showed the most significant enrichment. In the MF category, “estrogen response element binding” and “general transcription initiation factor binding” were the most highly enriched terms. (B) Bar plot summarizing the top enriched GO terms across the BP (blue), CC (red), and MF (green) categories. Bar length corresponds to the number of enriched genes (Count). BP terms such as “response to steroid hormone” and “nuclear receptor‐mediated signaling pathway” showed the highest gene counts. CC terms including “postsynaptic membrane” and “synaptic membrane” were prominently enriched. MF terms such as “estrogen response element binding” and “nuclear receptor activity” exhibited the highest enrichment frequency.

GO enrichment analysis of SC targets uncovered 25 significantly enriched terms across biological process (BP), cellular component (CC), and molecular function (MF) categories (Figure 3A,B). In BP, the terms “response to steroid hormone” (Gene Ratio = 0.087; adjusted *p* = 1.2 × 10^−5^) and “nuclear receptor‐mediated signaling pathway” (Gene Ratio = 0.073; adjusted *p* = 3.8 × 10^−5^) exhibited the highest enrichment significance and gene counts. In CC, “postsynaptic membrane” (Count = 18; adjusted *p* = 4.1 × 10^−5^) and “synaptic membrane” (Count = 15; adjusted *p* = 7.3 × 10^−4^) were most prominent. In MF, “estrogen response element binding” (Count = 12; adjusted *p* = 9.6 × 10^−4^) and “nuclear receptor activity” (Count = 10; adjusted *p* = 2.1 × 10^−4^) displayed the strongest enrichment. Bubble and bar plots consistently illustrated that steroid hormone‐related pathways and synaptic structural components were the dominant functional themes (Figure 3A,B), with all significant terms having adjusted *p* < 0.05.

### 2.4 SC Targets Significantly Enriched in Ribosomal, Infection‐Related, and ECM‐Receptor Interaction Pathways in Osteoarthritis

**FIGURE 4:**
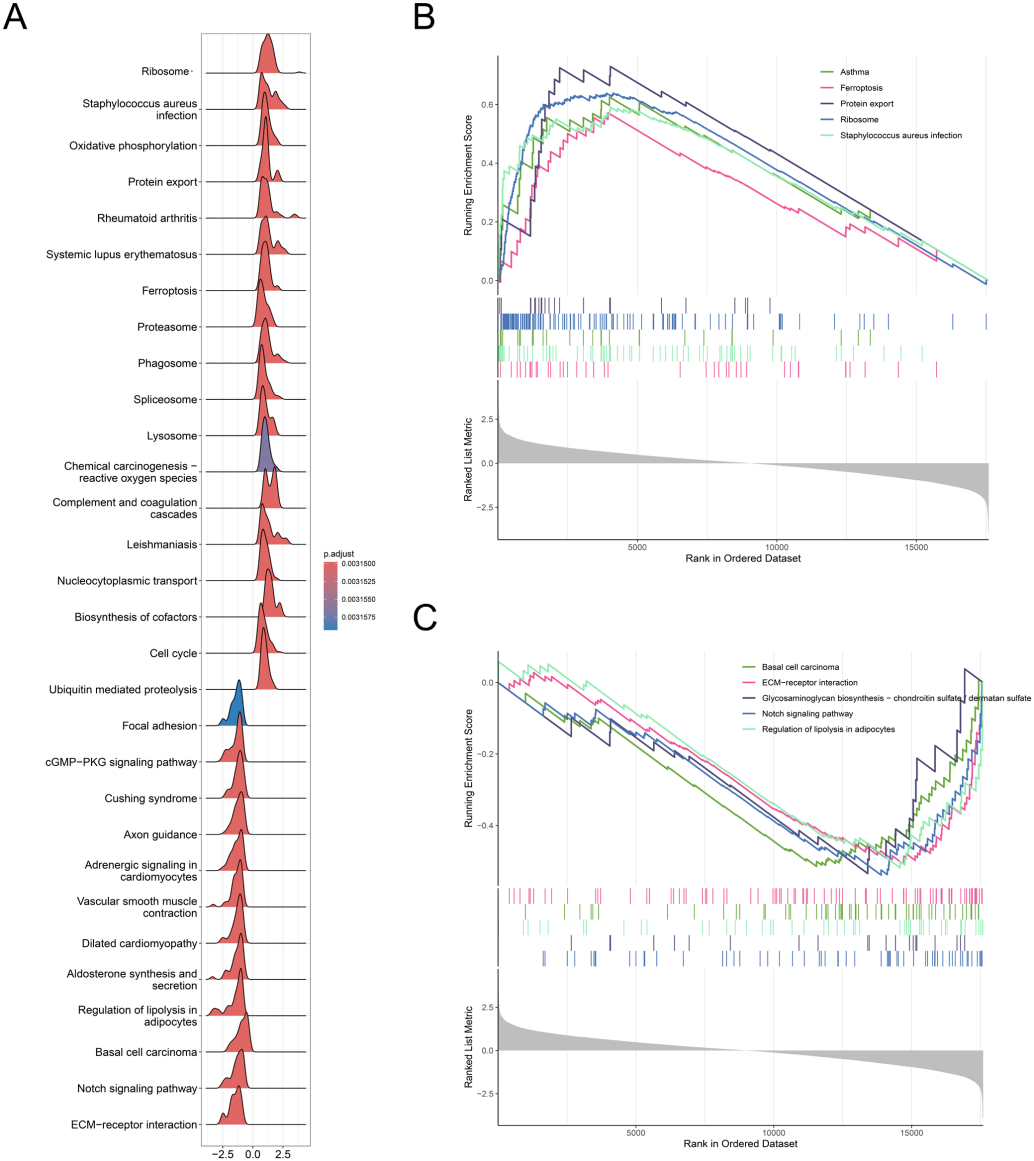
KEGG Pathway Enrichment and Gene Set Enrichment Analysis (GSEA) of *Schisandra chinensis* Targets in Osteoarthritis. (A) KEGG pathway enrichment analysis of *Schisandra chinensis* targets. The plot displays enrichment scores for 25 pathways, with pathway names on the *y*‐axis and enrichment scores on the *x*‐axis. A color gradient (from blue to red) represents adjusted *p*‐values (*p*.adjust), with darker red indicating higher statistical significance (smaller *p*.adjust values). Notably enriched pathways included “Ribosome,” “ *Staphylococcus aureus* infection,” and “Oxidative phosphorylation,” which showed strong enrichment signals (red peaks). The “Chemical carcinogenesis‐reactive oxygen species” pathway exhibited a distinct blue peak, indicating lower significance (*p*.adjust = 0.0031575). (B) GSEA enrichment plots for selected pathways, including “Asthma,” “Ferroptosis,” “Protein export,” “Ribosome,” and “ *Staphylococcus aureus* infection.” The upper panel shows the running enrichment score (*y*‐axis) across the ranked gene list (*x*‐axis), with peaks reflecting significant enrichment. The lower panel displays the ranked list metric, in which vertical bars indicate gene positions in the ranked dataset. Pathways such as “Ribosome” and “ *Staphylococcus aureus* infection” demonstrated prominent enrichment, characterized by steep curves and high peak scores, whereas “Ferroptosis” showed a moderate enrichment trend. (C) GSEA enrichment plots for additional pathways, including “Basal cell carcinoma,” “ECM‐receptor interaction,” “Glycosaminoglycan biosynthesis,” “Notch signaling pathway,” and “Regulation of lipolysis in adipocytes.” The upper panel illustrates the running enrichment score, and the lower panel shows the ranked list metric. Pathways such as “ECM‐receptor interaction” and “Notch signaling pathway” exhibited significant enrichment patterns, characterized by steep curves and concentrated gene distributions, suggesting their potential roles in osteoarthritis pathogenesis and the therapeutic mechanisms of *Schisandra chinensis*.

KEGG analysis identified 25 significantly enriched pathways (adjusted *p* < 0.05), with “Ribosome” (enrichment score = 1.82; adjusted *p* = 1.4 × 10^−8^), “ *Staphylococcus aureus* infection” (enrichment score = 1.65; adjusted *p* = 3.2 × 10^−5^), and “Oxidative phosphorylation” (enrichment score = 1.51; adjusted *p* = 2.7 × 10^−4^) demonstrating the highest statistical significance (Figure 4A). GSEA corroborated strong enrichment for “Ribosome” (normalized enrichment score [NES] = 2.15; *p* < 0.001) and “ *Staphylococcus aureus* infection” (NES = 1.98; *p* < 0.001), characterized by steep running score curves and concentrated leading‐edge gene distributions (Figure 4B). Additionally, “ECM‐receptor interaction” (NES = 1.87; *p* = 0.002) and “Notch signaling pathway” (NES = 1.79; *p* = 0.004) exhibited meaningful enrichment patterns (Figure 4C), while “Ferroptosis” showed only moderate enrichment (NES = 1.42; *p* = 0.03). Although the “Chemical carcinogenesis‐reactive oxygen species” pathway was enriched (adjusted *p* = 0.003), it displayed a weaker signal compared to other pathways (Figure 4A).

### 2.5 SC Active Compounds Formed Stable Hydrogen Bonds and Hydrophobic Interactions With PTGS2 at Key Binding Residues

**FIGURE 5:**
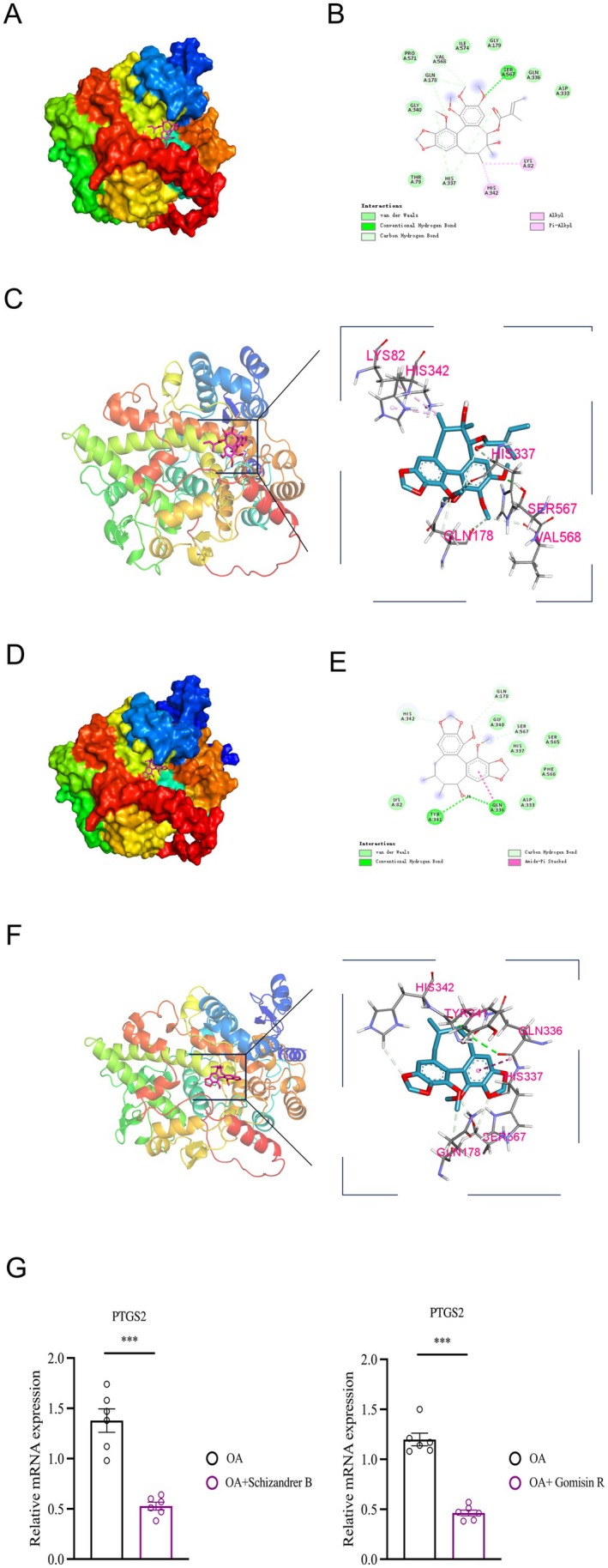
Molecular Docking and Binding Interaction Analysis of Active Compounds from *Schisandra* chinensis with Osteoarthritis‐Related Target Proteins. (A) PTGS2 and Schizandrer B. Three‐dimensional (3D) surface model of the target protein with color‐coded structural domains. The binding pocket is visualized with the docked active compound from *Schisandra chinensis* (magenta), highlighting the spatial conformation of the ligand within the protein cavity. (B) Two‐dimensional (2D) ligand‐protein interaction diagram of the compound bound to the target protein. Green dashed lines indicate hydrogen bonds with residues VAL343 and ALA524, while pink and blue shaded regions represent hydrophobic interactions and π‐alkyl interactions, respectively. (C) Molecular docking complex of the compound with the target protein. The left panel shows the overall protein structure, colored according to secondary structure elements, with the ligand (magenta) positioned in the binding site. The right panel provides a magnified view of the binding interface, labeling key interacting residues (LYS82, HIS342, GLN178, and VAL568) and their interaction types. (D) PTGS2 and Gomisin R. Three‐dimensional (3D) surface model of the target protein with domain‐specific color coding. The docked compound is shown within the binding pocket, demonstrating the spatial orientation of the ligand relative to the protein surface. (E) Two‐dimensional (2D) interaction diagram of the target protein, illustrating hydrogen bonds (green dashed lines) with residues GLY337 and SER336, as well as π‐π stacking interactions (pink shaded regions) with aromatic residues. (F) Molecular docking complex of the target protein. The left panel displays the protein‐ligand complex, whereas the right panel provides a magnified view of the binding site, highlighting key residues (HIS342, TYR341, GLN336, and SER367) involved in ligand binding through hydrogen bonds and hydrophobic interactions. (G) PTGS2 expression in the Schizandrer B and Gomisin R treatment groups.

Molecular docking analysis indicated that Schizandrer B, a compound of SC, exhibits high affinity binding to PTGS2, with a binding energy of −6.9 kcal/mol (Figure 5A–C). The 3D surface model confirmed the presence of compounds within the PTGS2 binding pocket (Figure 5A), while 2D analysis revealed hydrogen bonds with VAL343 and ALA524, along with hydrophobic/π‐alkyl interactions involving residues LY1522, HIS342, GLN178, and VAL568 (Figure 5B,C). Strong binding of PTGS2 to Gomisin R was also noted, exhibiting a binding energy of −8.4 kcal/mol. This interaction involved hydrogen bonding with GLY337 and SER336, π‐π stacking with aromatic residues, and hydrophobic contacts with HIS342, TYR341, GLN336, and SER367 (Figure 5D–F). All analyzed complexes demonstrated favorable binding conformations, with ligands deeply embedded within the target protein cavity and numerous residue‐specific interactions stabilizing the complexes. No spatial conflicts or unfavorable binding orientations were observed in any of the complexes analyzed. Additionally, qRT‐PCR results indicated a decrease in PTGS2 expression in the treatment groups receiving either Schizandrer B or Gomisin R (Figure 5G).

### 2.6 Molecular Dynamics Simulation of PTGS2 With Gomisin R and Schisandrin B

**FIGURE 6:**
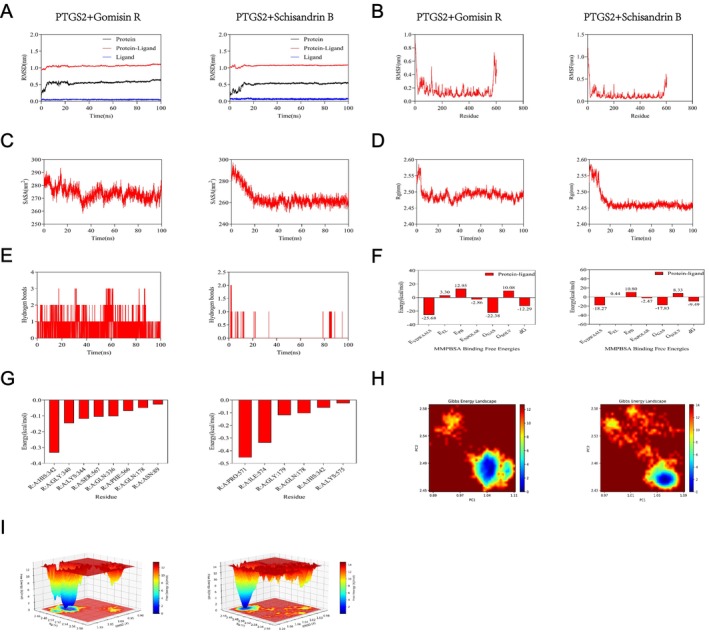
Molecular Dynamics Simulation. (A) RMSD of PTGS2 in complex with Gomisin R and Schisandrin B. (B) RMSF of PTGS2 in complex with Gomisin R and Schisandrin B. (C) SASA of PTGS2 in complex with Gomisin R and Schisandrin B. (D) Rg of PTGS2 in complex with Gomisin R and Schisandrin B. (E) Number of hydrogen bonds formed between PTGS2 and Gomisin R or Schisandrin B. (F) Binding free energy and its components for the PTGS2‐Gomisin R and PTGS2‐Schisandrin B complexes. (G) Energy contributions of key residues within the binding pocket to ligand binding. (H, I) Two‐dimensional (2D) and three‐dimensional (3D) free energy landscape plots of the PTGS2 complex with Gomisin R and Schisandrin B. Rg, radius of gyration; RMSD, root mean square deviation; RMSF, root mean square fluctuation; SASA, solvent‐accessible surface area.

To further assess the binding affinity between PTGS2 and Gomisin R, as well as Schisandrin B, molecular dynamics simulations were conducted. The root mean square deviation (RMSD) quantifies the conformational stability of proteins and ligands by measuring the deviation of atomic positions from their initial states; smaller deviations indicate enhanced stability (Coutsias and Wester 2019). RMSD serves as a metric for evaluating the stability of the simulation systems. As illustrated in Figure 6A, both complexes achieved equilibrium after 20 ns, confirming the stable binding of the drugs to PTGS2. The root mean square fluctuation (RMSF) assesses the flexibility of amino acid residues within proteins (Cao et al. 2024). Figure 6B demonstrates that the RMSF values for most residues in the core region remain low, suggesting stability in the conformation of the functional core region. Notably, a peak observed at the N‐terminus reflects the inherent flexibility characteristic of protein terminal regions, which does not significantly affect overall structural stability or the functional core. The solvent‐accessible surface area (SASA) is essential for evaluating protein surface exposure; this simulation analyzed the SASA between the target protein and small drug molecules, as shown in Figure 6C. Results indicate that the SASA values for both compounds exhibit steady fluctuations, signifying no substantial expansion or contraction post‐binding. The radius of gyration (Rg) illustrates structural changes and compactness of proteins, with larger Rg changes indicating system expansion. Figure 6D reveals that Rg values for both groups remained stable, without sustained increases or decreases throughout the process, indicating that the structural compactness persisted without unfolding or loosening, reinforcing that ligand binding led to increased protein compactness. To explore the properties of hydrogen bonds at the binding sites of the complex, the number of hydrogen bonds formed through stable interactions between the drug ligands and the protein was quantified. Figure 6E illustrates stable hydrogen bond interactions in both complexes, signifying sustained binding between the protein and the ligand. This stability provides a key force underpinning the persistent association of the ligand, further reinforcing the overall stability of the complex system. The MM‐PBSA calculations for the PTGS2‐Gomisin R complex indicate a binding free energy of −12.29 kcal/mol over the 0–100 ns period, reflecting strong binding between the two. Significant contributing residues include HIS: 342, GLY: 340, and LYS: 344 (Figure 6F,G). Similarly, the MM‐PBSA results for the PTGS2‐Schisandrin B complex reveal a binding free energy of −9.49 kcal/mol within the same timeframe, also demonstrating robust binding. Key residues contributing to this interaction include PRO: 571, ILE: 574, and GLY: 179 (Figure 6F,G). The 2D and 3D free energy profiles for both complexes indicate that the proteins predominantly occupy a dominant conformational state throughout the simulation, exhibiting a concentrated conformational distribution with minimal high‐energy discrete states. This suggests that the system experiences few conformational transitions, maintaining a stable thermodynamic state overall (Figure 6H,I). Collectively, these findings confirm the stability of PTGS2 binding to Gomisin R and Schisandrin B during molecular dynamics simulations and suggest potential biological activity.

### 2.7 Dynamic Characteristics of PTGS2 Expression in the Developmental Trajectory of Osteoarthritis

**FIGURE 7:**
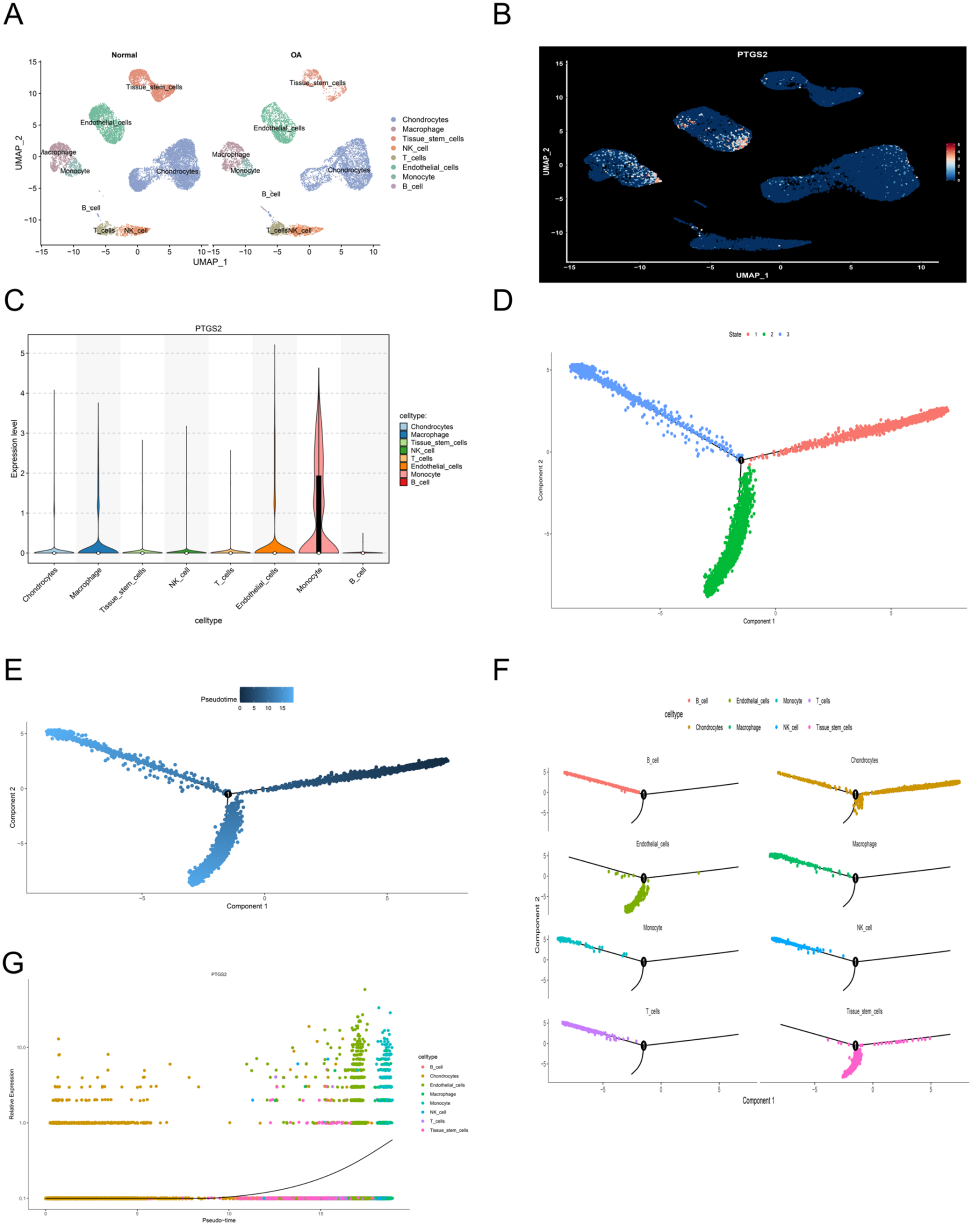
(A) UMAP visualization of single cells from Normal and OA samples. (B) UMAP plot showing PTGS2 expression. (C) Violin plot showing PTGS2 expression. (D) Cell trajectory state plot displaying the developmental trajectories of cells at different pseudotime points, with different cell states represented by different colors. (E) Pseudotime plot illustrating the simulated sequence of cell differentiation. (F) Pseudotime developmental trajectories of all subgroups. (G) Relative expression of PTGS2 across different pseudotime points.

Through UMAP‐optimized transcriptome profiling, the cellular landscape of two clinical cohorts, Normal and OA, was systematically deconstructed. Utilizing specific biomarkers for cell population segmentation, eight distinct cell subgroups were identified: Chondrocytes, B cells, Macrophages, Tissue stem cells, NK cells, T cells, Endothelial cells, and Monocytes (Figure 7A). Notably, PTGS2 expression was found to be associated with Monocytes and Endothelial cells (Figure 7B,C).

To simulate dynamic gene expression throughout OA progression, Monocle 2 was employed to perform trajectory analysis based on transcriptional alterations during cell differentiation. The findings delineated a transformation process comprising three distinct states, with the cell trajectory progressing from right to left (Figure 7D,E). Monocytes and endothelial cells were identified in the later stages of differentiation (Figure 7F), and PTGS2 expression exhibited a temporal increase (Figure 7G).

### 2.8 Cell Communication Analysis

**FIGURE 8:**
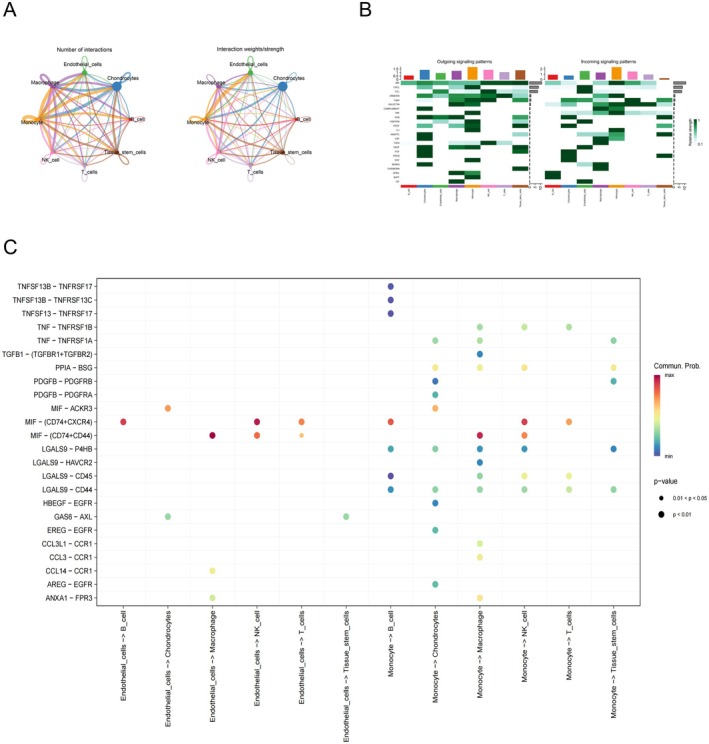
Cell Communication Analysis. (A) Communication network among different cell subpopulations, in which the line thickness on the left represents the number of receptors and the line thickness on the right represents the communication strength (weight). (B) Heatmap showing outgoing (left) and incoming (right) signaling patterns of different cell types. (C) Communication bubble plot comparing Monocytes and Endothelial cells with other cell types, showing differences in signaling interactions.

To elucidate intercellular communication dynamics within the OA microenvironment, the CellChat algorithm was implemented to construct and analyze ligand‐receptor interaction networks across various cell subgroups. This analysis revealed significant communication differences between Monocytes, Endothelial cells, and other cell types (Figure 8A,B). Interestingly, Endothelial cells and Monocytes engaged with NK cells and T cells through MIF − (CD74 + CXCR4) signaling, while MIF − (CD74 + CD44) signaling mediated interactions between Endothelial cells, Monocytes, NK cells, and Macrophages (Figure 8C).

### 2.9 Knockout of PTGS2 Can Identify Downstream Regulatory Networks

**FIGURE 9:**
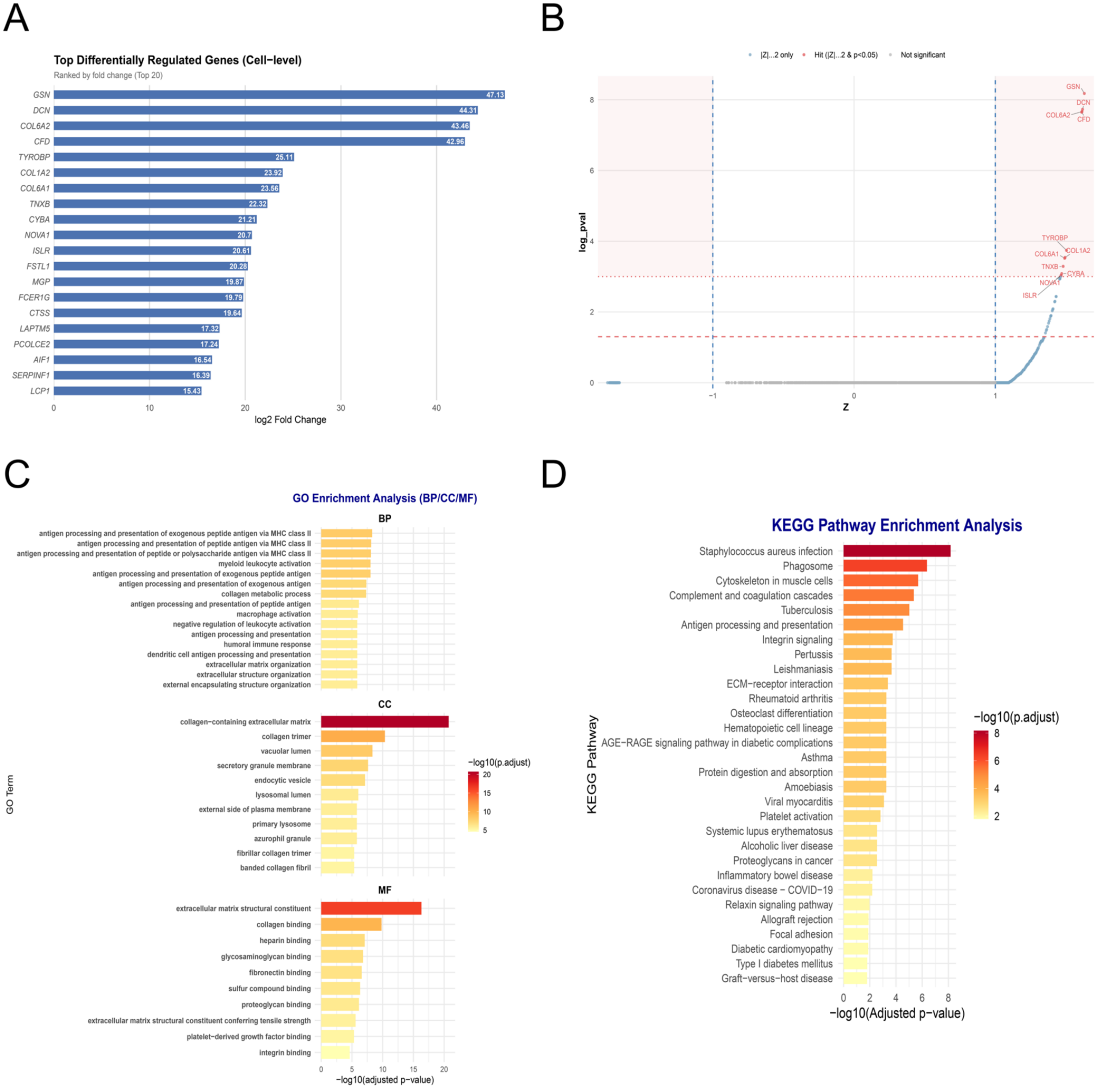
PTGS2 Knockout Identifies Downstream Regulatory Networks. (A, B) In silico knockout of PTGS2 in single cells revealed highly ranked differentially dysregulated genes. (A) Bar plot. (B) Scatter plot. (C) GO enrichment analysis. (D) KEGG pathway analysis.

To investigate the downstream effects of PTGS2 signaling and to identify potential effector pathways, computer gene knockout simulations using the scTenifoldKnk framework were conducted, eliminating regulatory connections within the inferred gene regulatory network from single‐cell data. The virtual knockout of PTGS2 revealed GSN as the most significantly affected downstream gene (Figure 9A,B). Pathway enrichment analysis indicated that the disrupted regulatory network strongly correlated with biological processes such as collagen‐containing ECM and structural components of the ECM (Figure 9C). Additionally, KEGG analysis revealed significant correlations between the knocked‐out regulatory network and pathways related to immune inflammation, ECM remodeling, cytoskeletal dynamics, and metabolic stress (Figure 9D).

## 3 Discussion

This study identified SC as a selective modulator of OA pathogenesis through integrated bioinformatics analysis. Venn diagram analysis revealed only five shared targets between SC and OA, indicating a targeted mechanism that may reduce off‐target effects compared to broad‐spectrum anti‐inflammatories. Among these targets, PTGS2 (COX‐2) emerged as the sole candidate with significant diagnostic potential (AUC = 0.702; *p* = 0.015), while AR, ESR1, DPP4, and CHRM2 demonstrated no clinical applicability. This finding aligns with existing clinical evidence. Prostaglandin endoperoxide synthase 2 (PTGS2), also known as cyclooxygenase‐2 (COX‐2), plays a critical role in the pathogenesis of various inflammation‐related diseases and tumors (Yu et al. 2016; Hashemi Goradel et al. 2019; Zheng et al. 2019). Previous clinical studies indicated that patients with knee OA exhibit significantly elevated COX‐2 levels compared to those with acute knee joint trauma, with marked increases in COX‐2 expression in synovial tissue and synovial fluid (Liu et al. 2021). Overexpression of PTGS2 can further amplify local inflammatory responses, enhance the production of multiple inflammatory factors, and exacerbate cartilage and joint structural damage (Cheng, Liu, et al. 2024). Thus, PTGS2 represents a biologically relevant molecular target closely linked to the inflammatory processes and clinical manifestations of OA.

Identifying PTGS2 as the sole diagnostically significant target (AUC = 0.702, *p* = 0.015) constitutes a notable advancement. Conventional non‐selective NSAIDs inhibit both COX‐1 and COX‐2 isoforms, resulting in gastrointestinal ulceration and cardiovascular complications. In contrast, molecular docking analyses demonstrate that SC bioactive compounds (MOL008957, MOL008978) bind specifically to the catalytic pocket of PTGS2. Hydrogen bonds with VAL343 and ALA524 anchor these compounds to key residues essential for prostaglandin synthesis: VAL343 positions arachidonic acid for oxygenation, while ALA524 coordinates the heme cofactor needed for cyclooxygenase activity. This binding mode resembles that of selective COX‐2 inhibitors (e.g., celecoxib) but avoids the cardiovascular risks associated with them by preserving COX‐1‐mediated cytoprotection in the gastric mucosa. The clinical significance is underscored by the consistent upregulation of PTGS2 in OA synovium and its established role in driving synovitis and pain progression. Thus, SC offers therapeutic selectivity without the safety compromises associated with current treatment options.

Additionally, SC is significantly enriched in the “ECM‐receptor interaction” pathway (NES = 1.87, *p* = 0.002), which is directly implicated in the structural progression of OA. Data suggest that SC compounds may help preserve cartilage integrity through modulation of PTGS2‐dependent signaling. COX‐2‐derived prostaglandins upregulate matrix metalloproteinases (MMPs)—notably MMP‐13—within chondrocytes while suppressing integrin‐mediated ECM adhesion. By selectively inhibiting PTGS2, SC could disrupt this deleterious cycle, preventing the degradation of collagen and aggrecan. This mechanism fundamentally diverges from NSAIDs, which tend to exacerbate cartilage damage via COX‐1 inhibition and fail to halt structural decline. The synthesis of PTGS2 inhibition and ECM stabilization positions SC as a distinctive candidate for the development of a true DMOAD, effectively addressing both inflammation and joint destruction.

The limited number of shared targets (AR, ESR1, DPP4, CHRM2, PTGS2) may initially seem inconsistent with the phytochemical complexity of SC. However, this observation reflects biological filtering rather than a mechanistic limitation. Rigorous diagnostic validation excluded non‐dysregulated targets; for instance, ESR1 exhibited no significant expression differences (*p* = 0.9; AUC = 0.511), consistent with its heterogeneous expression across joint tissues. Similarly, AR (*p* = 0.41; AUC = 0.570) and CHRM2 (*p* = 1.0; AUC = 0.501) demonstrated a lack of pan‐OA diagnostic utility. Molecular docking, however, confirmed stable binding of SC compounds to PTGS2, indicating context‐dependent efficacy in subsets where androgen signaling may contribute to the severity of OA. This finding supports stratified therapeutic strategies, such as using SC for male patients with heightened synovial androgen activity. Furthermore, functional enrichment in “response to steroid hormone” (adjusted *p* = 1.2 × 10^−5^) integrates all five targets into a cohesive anti‐inflammatory‐catabolic signaling axis, as glucocorticoid dysregulation in OA upregulates both PTGS2 and MMPs.

Single‐cell transcriptome analysis revealed eight distinct cell subpopulations in normal and OA joint tissues. Notably, PTGS2 expression was predominantly observed in monocytes and endothelial cells, with a stronger presence in monocytes. As a critical component of the innate immune system, monocytes play a significant role in OA pathogenesis (Gómez‐Aristizábal et al. 2019). Under inflammatory conditions associated with OA, monocytes frequently migrate to the subchondral bone region, where they differentiate into osteoclasts and macrophages (Zhu et al. 2019). In addition, these monocytes secrete inflammatory factors, intensifying the OA inflammatory response and contributing to cartilage degeneration (Rezuș et al. 2019). Trajectory analysis using Monocle 2 demonstrated that monocytes are in a late stage of differentiation, with PTGS2 expression gradually increasing over time. This temporal expression pattern signifies that PTGS2 serves as a hallmark of pro‐inflammatory effector cells undergoing terminal differentiation. Cell communication analysis revealed that the MIF − (CD74 + CXCR4) and MIF − (CD74 + CD44) signaling pathways represent key interaction axes linking endothelial cells and monocytes with NK cells and T cells. Macrophage migration inhibitory factor (MIF) is an inflammatory cytokine noted for its role in modulating immune responses, acting through two distinct receptor complexes: MIF‐CD74 + CD44 and MIF‐CD74 + CXCR4 (Tu et al. 2025). The findings suggest that SC may indirectly disrupt MIF‐dependent intercellular communication networks by downregulating PTGS2, thereby attenuating the recruitment and activation of monocytes within the OA microenvironment.

Finally, scTenifoldKnk was utilized to virtually knock out PTGS2, identifying GSN as the most significantly affected downstream gene. GSN, an actin‐binding protein, is implicated in cell movement, shape, metabolism, and wound healing processes (Feldt et al. 2019; Wittmann et al. 2018). It is produced by macrophage‐like synovial cells (MLS) and fibroblast‐like synovial cells (FLS) in the synovium and secreted into the synovial fluid. Previous studies have demonstrated a significant reduction of GSN levels in the synovial fluid of patients with OA (Feldt et al. 2022). GSN is known to exert protective effects on chondrocytes, facilitating cartilage regeneration and differentiation (Feldt et al. 2020; Chan et al. 2019). Therefore, the presence of GSN in synovial fluid is essential for maintaining the health of cartilage and joints. Pathway enrichment analysis of the PTGS2 knockout regulatory network highlighted “ECM‐containing collagen,” “ECM structural components,” “immune inflammation,” “ECM remodeling,” “cytoplasmic cytoskeleton dynamics,” and “metabolic stress‐related pathways.” This pattern highlights PTGS2 as a key regulator coordinating matrix degradation, immune cell infiltration, and cytoskeletal dysfunction in OA. Identifying GSN as a primary downstream candidate paves the way for further understanding the cartilage‐protective effects of PTGS2 inhibition.

Several limitations warrant careful consideration. First, future research should validate the effects of PTGS2 on ECM in chondrocytes and subchondral bone. Second, while molecular docking confirms binding affinity to PTGS2, in vitro assays are necessary to quantify the inhibitory potency (IC_50_) of individual SC compounds (Patel et al. 2008; Wang, Li, Wang, et al. 2024). Third, PTGS2's moderate diagnostic AUC (0.702) indicates that the incorporation of combinatorial biomarkers (e.g., PTGS2 + COMP fragments) may enhance the accuracy of patient stratification (Schichlein et al. 2025). Fourth, the robust enrichment of the ribosomal pathway (adjusted *p* = 1.4 × 10^−^8), potentially associated with chondrocyte senescence, requires experimental validation to establish its causal relevance to the mechanisms of SC (Mukherjee and Das 2024). Fifth, the analyses of cell‐to‐cell communication and regulatory networks using scTenifoldKnk are based on computational inferences, particularly concerning predicted interactions involving MIF‐CD74/CXCR4, MIF‐CD73/CD44, and alterations in GSN. Future studies should conduct experiments on larger sample sizes to validate these causal relationships and regulatory mechanisms. Sixth, while our qRT‐PCR data indicated a decrease in PTGS2 expression following treatment with Schizandrin B and Gomisin R, we did not assess the cytotoxicity of these compounds, their selectivity for PTGS1, or their in vivo efficacy in OA animal models.

These findings have three immediate clinical implications. First, SC's selectivity for PTGS2 provides a framework for developing next‐generation COX‐2 inhibitors with enhanced safety profiles, which is particularly critical for elderly patients with OA facing the risks of polypharmacy (Shi et al. 2025). Second, the dual targeting of both inflammation (PTGS2) and structural integrity (ECM) positions SC as a promising candidate for DMOAD, addressing a critical unmet need in OA therapeutics (Shu et al. 2023; Wang, Li, Wu, et al. 2024). Third, synovial PTGS2 expression could serve as a stratification biomarker to identify “SC‐responsive” patients, specifically those with early inflammatory OA, thereby advancing precision medicine in musculoskeletal disorders (Muthumalage and Rahman 2025).

## 4 Conclusion

In summary, this study performed a comprehensive systematic pharmacological analysis of SC in OA, identifying PTGS2 as the primary therapeutic target, with monocytes serving as the key site of PTGS2 action. Future research should focus on experimental validation of PTGS2, optimization of SC‐derived compounds with improved PTGS2 selectivity, and evaluation of their efficacy in post‐traumatic and age‐related OA models.

## 5 Materials and Methods

### 5.1 Collection of *Schisandra chinensis* Active Compounds

The chemical constituents of SC were sourced from the Traditional Chinese Medicine Systems Pharmacology Database and Analysis Platform (TCMSP, https://tcmspw.com/tcmsp.php). Selection criteria for the constituents included oral bioavailability (OB) of ≥ 30% and drug‐likeness (DL) of ≥ 0.18, as these parameters are widely recognized thresholds for predicting compound bioavailability and pharmacokinetic properties. Additionally, compounds were required to possess a molecular weight between 180 and 500 Da, an octanol–water partition coefficient (logP) ≤ 5, a maximum of five hydrogen bond donors, and a maximum of ten hydrogen bond acceptors to comply with Lipinski's Rule of Five. The 3D structures of the selected compounds were downloaded in SDF format from the PubChem database (https://pubchem.ncbi.nlm.nih.gov/) for subsequent molecular docking analysis.

### 5.2 Osteoarthritis‐Related Target Collection

OA‐associated genes were systematically gathered from five authoritative databases. The Online Mendelian Inheritance in Man (OMIM, https://omim.org/) was searched using the term “osteoarthritis,” with manual curation of disease‐associated genes. The Therapeutic Target Database (TTD, http://db.idrblab.net/ttd/, version 2023) provided additional OA‐related targets through disease annotation. GeneCards was utilized to eliminate duplicate targets using the unique function in R (version 4.2.2), with gene symbols standardized against the UniProt database (https://www.uniprot.org/).

### 5.3 Target Intersection Analysis

The intersection of potential targets from SC and OA‐related genes was visualized using a Venn diagram generated by the VennDiagram package (version 1.7.1) in R (Meng‐ran et al. 2020). A five‐way intersection analysis identified common targets across all databases, which were designated as core therapeutic targets for further analyses.

### 5.4 Protein–Protein Interaction (PPI) Network Analysis

Based on the previously identified five core objectives, a PPI network was constructed using the STRING database (https://string‐db.org/, version 11.5), limiting the species to *Homo sapiens* and setting a confidence threshold > 0.7. The resulting network file was then imported into Cytoscape (version 3.9.1) for visualization and topological analysis. Node degree, betweenness centrality, and closeness centrality were calculated using the NetworkAnalyzer plugin to identify hub targets.

### 5.5 Differential Expression and Diagnostic Value Analysis

Gene expression profiles from patients with OA and healthy controls were obtained from the Gene Expression Omnibus (GEO) database (accession number: GSE89408), which includes 28 normal samples and 22 OA samples sourced from human synovial biopsies. Raw data were normalized using the Robust Multi‐array Average (RMA) algorithm in R, followed by batch effect correction using ComBat. Differential expression analysis was conducted using the limma package (version 3.54.0), applying significance thresholds of |log_2_FC| > 0.5 and adjusted *p*‐value < 0.05. ROC curve analysis was performed using the pROC package (version 1.18.0) to evaluate diagnostic efficacy, with an AUC > 0.65 considered indicative of moderate diagnostic value.

### 5.6 Gene Ontology (GO) Functional Enrichment

GO enrichment analysis was performed for BP, CC, and MF using the clusterProfiler package (version 4.6.0) in R. This analysis included all five core targets and their first‐order interactors (totaling 52 genes) retrieved from STRING. Homo sapiens was specified as the organism, and the Benjamini‐Hochberg method was used to adjust *p*‐values, setting the threshold for statistical significance at an adjusted *p* < 0.05 (Li et al. 2013). The top 20 enriched terms in each category were visualized through bubble plots and bar plots created using ggplot2 (version 3.4.0).

### 5.7 KEGG Pathway Enrichment and Gene Set Enrichment Analysis (GSEA)

KEGG pathway enrichment analysis was performed using the clusterProfiler package, applying the same significance threshold of adjusted *p* < 0.05 (Arnold et al. 2025). To complement this analysis, GSEA was conducted on the entire gene expression profile from GSE114007, which included 40 patients with OA and 10 controls. Utilizing the clusterProfiler and fgsea packages, genes were ranked by signal‐to‐noise ratio, and 1000 permutations were performed to calculate NES and false discovery rate (FDR) *q*‐values. Pathways exhibiting |NES| > 1.5 and FDR *q*‐values < 0.25 were considered significantly enriched, adhering to standard GSEA interpretation guidelines.

### 5.8 Protein Structure Preparation

The crystal structures of PTGS2 (PDB ID: 5KIR) and AR (PDB ID: 2PIU) were downloaded from the Protein Data Bank (https://www.rcsb.org/). Water molecules and co‐crystallized ligands were removed using AutoDock Tools (version 1.5.7), and polar hydrogen atoms were added (Zheng et al. 2019). The protein structures were subjected to energy minimization using the AMBER99 force field in UCSF Chimera (version 1.17), employing 100 steps of steepest descent followed by 100 steps of conjugate gradient minimization.

### 5.9 Ligand Preparation and Molecular Docking

The 3D structures of active compounds from SC (MOL008957, MOL008978) were prepared by adding hydrogen atoms, assigning Gasteiger charges, and performing energy minimization using the MMFF94 force field in Open Babel (version 3.1.1). Molecular docking was executed using AutoDock Vina (version 1.2.0), with the binding site defined as a 20 Å × 20 Å × 20 Å grid centered on the coordinates of the native ligand. The exhaustiveness parameter was set to 50 to ensure thorough conformational sampling. The best docking pose was chosen based on the lowest binding energy (kcal/mol), and results were visualized using PyMOL (version 2.5.2) and LigPlot+ (version 2.2).

### 5.10 Molecular Dynamics Simulation

Molecular dynamics simulations of the protein‐ligand complexes were conducted using Gromacs 2025. Proteins were processed using AMBER14SB force field parameters, while ligand topology files were constructed using GAFF2 force field parameters. The topology files for both the protein and ligand were merged, and the atomic types and related parameters were checked to prevent conflicts or deletions. The simulation system utilized periodic boundary conditions (PBC), with the protein‐ligand complex placed within a cubic water box. A minimum distance of 1.2 nm was maintained between the surface of the complex and the box wall, and the TIP3P water model was used for solvation. The total system charge was neutralized by adding appropriate Na + ions. Energy minimization was performed via the steepest descent method, followed by both NVT (isothermal and isochoric) and NPT (isothermal and isobaric) equilibrations, each consisting of 100,000 steps, with coupling constants of 0.1 ps and 100 ps durations. The final production simulation included 50,000,000 steps, each with a time step of 2 fs, resulting in a total simulation time of 100 ns. Following the simulation, trajectory analysis was executed using built‐in tools within the software, calculating metrics such as RMSD, RMSF, SASA, and protein rotation radius. Free energy calculations (MMGBSA), free energy landscapes, and other relevant data were also obtained.

### 5.11 Single Cell Data Analysis

Strict quality control measures were applied to the single‐cell transcriptome data obtained from the GSE216651 dataset to ensure accurate representation of cell status and gene expression patterns. Cells exhibiting fewer than 200 or more than 10,000 gene detections, as well as those with mitochondrial gene expression exceeding 20%, were excluded to eliminate low‐quality or doublet cells. After filtering, the “NormalizeData” function from the Seurat package was utilized for data standardization, and the “FindVariableFeatures” function identified highly variable genes to capture transcriptome diversity (Satija et al. 2015). Subsequently, principal component analysis (PCA) was performed to determine the optimal number of principal components, guided by ElbowPlot and JackStrawPlot. Clustering was executed using the “FindClusters” function with a resolution of 1.5, followed by UMAP dimensionality reduction with “RunUMAP” to visualize distinct cell populations. Cluster‐specific marker genes were identified using the “FindAllMarkers” function, and cell type annotation was performed based on established human cell markers utilizing the “SingleR” package, leading to the construction of a detailed map of the tumor microenvironment (Aran et al. 2019).

### 5.12 Pseudo Time Trajectory Analysis

In the pseudo time trajectory analysis, Monocle 2 was employed to infer differentiation trajectories of various cell subpopulations (Cao et al. 2023). Initially, a CellDataset was constructed from highly variable genes, and dimension reduction was achieved using the DDRTree method. A developmental trajectory was then established via the Minimum Spanning Tree (MST). Cell states and their distribution in pseudo time were visualized using the plot_CellTrajectory function, followed by BEAM analysis to identify branch‐specific genes. This was integrated with functional enrichment analysis to explore the biological functions associated with the trajectory.

### 5.13 Cell Communication Analysis

Cell communication between various cell subpopulations was analyzed using the CellChat software package. The standardized gene expression matrix was imported into CellChat to construct a communication network. Overexpression genes and their interactions were identified through the “identify overexpression genes” and “identify overexpression interactions” functions. The computeCommunProb and filterCommunication functions were employed to predict potential ligand‐receptor interactions, and the aggregateNet function was finally used to visualize the communication network.

### 5.14 Key Gene Knockout Analysis

Virtual knockout of key genes was performed using scTenifoldKnk (version 1.0.1) to identify downstream regulatory targets (Osorio et al. 2022). Gene regulatory networks were inferred from single‐cell data through bootstrap resampling (bootstrap = 10) and regression‐based edge estimation. Network topology was compared before and after the virtual knockout to quantify perturbation effects, with statistical significance evaluated via permutation testing and FDR correction.

### 5.15 Quantitative Real‐Time Polymerase Chain Reaction (qRT‐PCR)

qRT‐PCR analysis was organized into two groups: an OA group and an OA + Schizandrin B group, as well as an OA group and an OA + Gomisin R group. Each group comprised six biological replicates. Total RNA was extracted using the TRIzol method, and qRT‐PCR was then conducted on the LightCycler 480 real‐time fluorescence quantitative PCR system (Roche, USA) employing FastStart Universal SYBR Green Master. Two micrograms of RNA from each sample were utilized for reverse transcription, with a reaction volume of 20 μL that included 2 μL of cDNA template, 10 μL of PCR mixture, 0.5 μL of forward and reverse primers each, and the remainder made up with deionized water. The PCR program was initiated with an initial denaturation step at 95°C for 30 s, followed by 45 cycles consisting of 15 s of denaturation at 94°C, 30 s of annealing at 56°C, and 20 s of extension at 72°C. Each sample underwent six replicates. The cyclic threshold (Ct) data were calculated using the 2^−ΔΔCt^ method and normalized against GAPDH as an internal reference for each sample. The primer sequences for the target genes are provided in Table 1.

**TABLE 1:** Primer pair sequences for the target genes.

Gene names	Forward primer	Reverse primer
PTGS2	CGGTGAAACTCTGGCTAGACAG	GCAAACCGTAGATGCTCAGGGA

### 5.16 Statistical Analysis

All statistical analyses were conducted using R (version 4.2.2). Continuous variables are presented as mean ± standard deviation (SD). Group comparisons were performed using Student's *t*‐test for normally distributed data, while the Mann–Whitney *U* test was utilized for non‐normally distributed data, as assessed by the Shapiro–Wilk test. A *p*‐value of < 0.05 was considered statistically significant, with adjustments made for multiple comparisons where applicable. ROC curve analysis was performed based on DeLong's method to compare AUC values.

## Author Contributions

**Cheng Chen:** conceptualization, writing – review and editing, funding acquisition. **Yikai You:** methodology, software, writing – original draft. **Shiqi Ren:** data curation, formal analysis, validation, writing – review and editing. **Lingtian Min:** conceptualization, writing – original draft.

## Funding

This study was supported by Suqian Sci&Tech Program (Grant No. K202310), Medical Research Foundation of Jiangsu Provincial Health Commission (No. Z2023045), Suqian First Hospital “136” Youth Funding Project and Nanjing Medical University Sci&Tech Development Fund (NMUB20210290), Clinical Medicine Special Program of Nantong University (2024JZ038).

## Ethics Statement

The authors have nothing to report.

## Conflicts of Interest

The authors declare no conflicts of interest.

## Data Availability

All public data were obtained from the GEO database (https://www.ncbi.nlm.nih.gov/geo/).
